# Factors behind the success story of under-five stunting in Peru: a district ecological multilevel analysis

**DOI:** 10.1186/s12887-017-0790-3

**Published:** 2017-01-19

**Authors:** Luis Huicho, Carlos A. Huayanay-Espinoza, Eder Herrera-Perez, Eddy R. Segura, Jessica Niño de Guzman, María Rivera-Ch, Aluisio J.D. Barros

**Affiliations:** 1 Centro de Investigación para el Desarrollo Integral y Sostenible, Universidad Peruana Cayetano Heredia, Av. Honorio Delgado 430, LI33 Lima, Peru; 2 School of Medicine, Universidad Peruana Cayetano Heredia and Universidad Nacional Mayor de San Marcos, Lima, Peru; 3 Instituto Nacional de Salud del Niño, Lima, Peru; 4 School of Medicine, Universidad Peruana de Ciencias Aplicadas, Lima, Peru; 5 Ministerio de Economía y Finanzas, Lima, Peru; 6 Facultad de Ciencias y Filosofía, Universidad Peruana Cayetano Heredia, Lima, Peru; 7 International Center for Equity in Health, Federal University of Pelotas, Pelotas, Brazil

**Keywords:** Children, Stunting, Social determinants, Economic growth, Poverty, Childhood interventions, Ecologic study, Multilevel mixed-effects analysis

## Abstract

**Background:**

Stunting prevalence in children less than 5 years has remained stagnated in Peru from 1992 to 2007, with a rapid reduction thereafter. We aimed to assess the role of different predictors on stunting reduction over time and across departments, from 2000 to 2012.

**Methods:**

We used various secondary data sources to describe time trends of stunting and of possible predictors that included distal to proximal determinants. We determined a ranking of departments by annual change of stunting and of different predictors. To account for variation over time and across departments, we used an ecological hierarchical approach based on a multilevel mixed-effects regression model, considering stunting as the outcome. Our unit of analysis was one department-year.

**Results:**

Stunting followed a decreasing trend in all departments, with differing slopes. The reduction pace was higher from 2007–2008 onwards. The departments with the highest annual stunting reduction were Cusco (−2.31%), Amazonas (−1.57%), Puno (−1.54%), Huanuco (−1.52%), and Ancash (−1.44). Those with the lowest reduction were Ica (−0.67%), Ucayali (−0.64%), Tumbes (−0.45%), Lima (−0.37%), and Tacna (−0.31%). Amazon and Andean departments, with the highest baseline poverty rates and concentrating the highest rural populations, showed the highest stunting reduction. In the multilevel analysis, when accounting for confounding, social determinants seemed to be the most important factors influencing annual stunting reduction, with significant variation between departments.

**Conclusions:**

Stunting reduction may be explained by the adoption of anti-poverty policies and sustained implementation of equitable crosscutting interventions, with focus on poorest areas. Inclusion of quality indicators for reproductive, maternal, neonatal and child health interventions may enable further analyses to show the influence of these factors. After a long stagnation period, Peru reduced dramatically its national and departmental stunting prevalence, thanks to a combination of social determinants and crosscutting factors. This experience offers useful lessons to other countries trying to improve their children’s nutrition.

**Electronic supplementary material:**

The online version of this article (doi:10.1186/s12887-017-0790-3) contains supplementary material, which is available to authorized users.

## Background

Stunting in under-five children has been established as a risk factor for reduced intellectual performance and job productivity in later life, particularly when this stunting occurs in the first 2 years of life [1]. Hence global and country level efforts have been directed to develop policies and programmes aimed at effective reduction in the number of stunted children, with changing emphasis from food security to multisectoral approaches [2–6].

Stunting prevalence in children less than 5 years remained stagnant in Peru at around 30-40% from 1992 to 2007, with a rapid reduction thereafter [7]. This trend occurred within a context of heavy emphasis on implementation of food assistance interventions during the 1990s and the early 2000s [8, 9], which were not necessarily targeted to infants and pre-school children.

Although significant efforts have been made to explain the reasons for the initial lack of impact of the interventions and the underlying factors driving the more recent success story in Peru at national level [3, 8–10], we are not aware of systematic studies addressed to assess potential influential factors at district level.

To overcome the caveat of analyses limited to the national level that hide sub-national disparities, we aimed to assess the role of different predictors on reduction of under-five stunting over time and across districts (departments) in Peru, during the period 2000 to 2012. This study is part of a wider Countdown to 2015 Country Case Study, which is reported elsewhere [11]. We aimed at assessing predictors associated with departmental level prevalence of stunting.

## Methods

### Conceptual framework

**Fig. 1:**
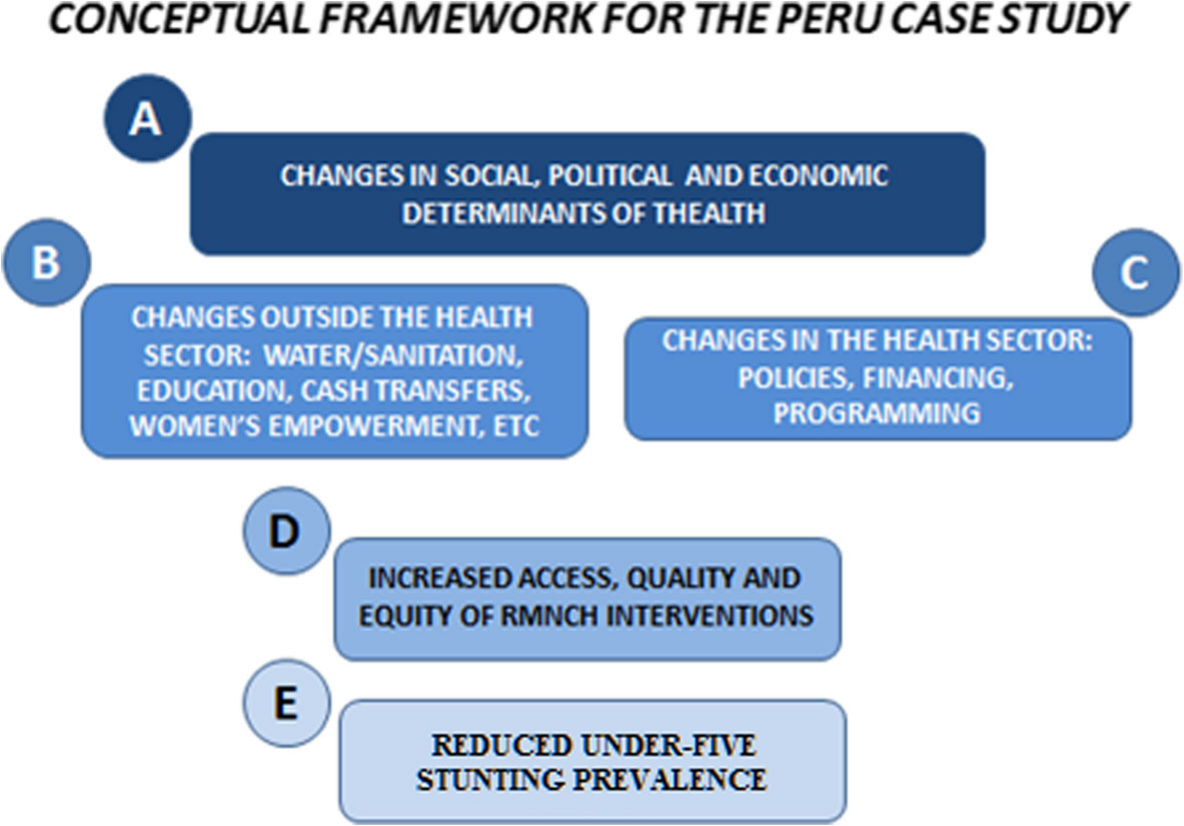
Conceptual framework of different levels of variables, from distal to proximal determinants of under-five stunting. RMNCH: Reproductive, maternal, newborn and child health. Re-used from reference [11]

A conceptual framework was developed to guide our analyses, and it considers different hierarchical variables, ranging from distal to proximal determinants of stunting (Fig 1) [11]. It includes four dimensions of factors that could explain departmental level variation of stunting during the study period, namely: a) social determinants (gross domestic product (GDP) per capita in constant 2012 US$, Gini for income index, percentage of families with at least one unmet basic need, percentage of families living under the poverty line, percentage or urban population, b) out-of-health sector changes (median years of women’s schooling, total fertility rate, percentage of households with piped water inside the house, rural coverage, rural coverage of JUNTOS conditional cash transfer programme-families per 10,000 rural population), c) health sector changes (utilisation of the Comprehensive Health Insurance System SIS-number of annual under-five outpatient preventative and clinical attendances per total under-five population; density of human resources of health, defined as the number of doctors, nurses and midwives per 10,000 population; per capita expenditure on child health in constant 2012 US$), d) composite coverage index-CCI (a weighted mean of the coverage of eight preventive and curative interventions covering four steps of the continuum of care that includes family planning, maternity care, child immunization, and case management) [12]. The impact indicator was under-five stunting prevalence, defined as the percentage of children younger than 5 years whose height or length for age was below 2 SD from the median, according to the World Health Organization standards.

### Data sources

We used different data sources for gathering information on our different study variables at departmental level. For stunting prevalence and coverage of reproductive, maternal, neonatal and child health (RMNCH) interventions potentially relevant to stunting prevalence evolution, we used the Peruvian Demographic and Health Information surveys (DHS) [7]. We used data from DHS for years 2000, 2004, 2005, 2006, 2007, 2008, 2009, 2010, 2011, and 2012. Peru did not conduct DHS in 2000, 2001 and 2002. Data on GDP per capita and percentage of urban population were obtained from the National Institute of Statistics and Computing [13, 14]. Percentage of households living under the poverty line and percentage of households with at least one unmet basic need were derived from the National Household Surveys [15]. Conditional cash transfer programme JUNTOS coverage (number of families beneficiaries of JUNTOS per 1,000 rural population) was extracted from the programme official website [16]. The number of under-five attendances by total under-five population for the Comprehensive Health Insurance System (SIS) were obtained from its website [17]. Density of doctors, nurses and midwives per 10,000 population was estimated from the data provided by the corresponding Ministry of Health website [18]. Further information on JUNTOS and SIS is provided elsewhere [11, 19].

### Time trends

We described time trends of main variables at departmental level, including stunting prevalence and each predictor variable from our conceptual framework.

### Ranking of departments by annual change of different variables

We estimated a ranking of departments according to their annual change of stunting prevalence over the study period, through linear regressions against time (year). Similar rankings were determined according to the annual change of all predictor variables considered in our analyses.

We additionally contrasted variation of the stunting prevalence by department through comparison of years 2000 and 2012.

### Departmental level mixed-effects analyses

#### Handling of missing data: imputation

The vast majority of study variables had no missing values. We had incomplete data for density of doctors, nurses and midwives for years 2000, 2005, 2008 and 2011, due to lack of consistently and reliably collated information from the official source. As for household access to improved water sources, the annual available DHS datasets had missing data for year 2004. Thus we imputed those missing values, through regression-based and tree-based imputation methods [20, 21]. For regression-based imputation, simple linear regressions of the variable of interest against time (year) were run [20]. SAS Enterprise Miner 4.3 was used to develop tree-based imputations with surrogates for missing class and interval variables [21]. The regression and imputed variables were consistent, and thus only one of them (regression-based) was used in the ecological analyses.

#### Stepwise mixed-effects linear regression

We attempted to account for the influence of the different variables included in the different dimensions of our conceptual framework over time and across departments [22]. Therefore we chose a stepwise multilevel mixed-effects linear regression model, with department- level prevalence of stunting for each study year as our unit of analysis. We used a simple variance multilevel model with one variance term for the measures at each time point (level 1) and one variance term for the department level (level 2). This model takes into account the fixed effects of the predictors (structured in our conceptual framework) and also the fixed effects of time. The random effects take into account the variability within departments, over time, as well as between departments [23]. The general formulation of this model is *y*_*ij*_ = ∑_*k* = 0_^*p*^ *β*_*k*_ *x*_*k*_ + *u*_*j*_ + *eij*, where y_ij_ is the prevalence of stunting in time i, department j, the βs are the fixed effects for the predictors and u_j_ and e_ij_ are the variability terms between and within departments, respectively.

The modeling was done at departmental level, because the individual modeling, despite theoretical advantages, is strongly driven by individual characteristics, specially poverty. This is not so well measured in surveys and ends up giving way to residual confounding, so that any determinant that also marks poverty ends up as a predictor of stunting. This is the case, for instance, of the percent of population covered by a cash transfer program – poorer areas have a higher percentage, and the program ends up a risk factor for stunting, not a preventive factor.

We aimed at using a parsimonious model. The model variables within each box from A to E were selected from our general conceptual framework. They were chosen on the basis of the available evidence about their influence on stunting. We additionally excluded a priori variables with similar characteristics, to avoid multicollinearity. For example, instead of including both variables related to the percentage of households living under the poverty line and the percentage living under the extreme poverty line, we retained only poverty line. Similarly, instead of including both variables related to the percentage of households with at least one unmet basic need and the percentage with four unmet basic needs, we retained only the percentage with one unmet need.

For each box of our conceptual model, starting with box A, we first run crude mixed-effects linear regressions, with stunting and one predictor at a time. We selected first those variables with *p* < = 0.20 irrespective of their direction [24], to run an adjusted multi-level mixed-effects linear regression with time as a locked term, that is a variable included in the model irrespective of the selection criteria. Our model includes a variable year that models the time changes. In the final model, it is not significant, which means that our predictors are capable of explaining the changes over time observed with stunting. We performed then a backward stepwise exclusion of variables with *p*-values higher than 0.20, starting with that with the highest *p*-value. We obtained in this way a final model for each box, with variables that had to be incorporated in the final models of the subsequent boxes.

We repeated the crude regressions of the outcome with each predictor in box B, as well as the backward stepwise selection for variables with *p* < =0.20. Then the final selected variables in box B and the final selected variables from box A were run together in a new multivariate model, which was followed by a new backward stepwise selection, to obtain the final model for box A plus B. The variables of this final model were kept for incorporation in the final models of the next boxes.

We repeated the same steps with box C and box D, incorporating the selected variables from previous boxes. In all cases, variables already included from previous boxes were kept until the final model, regardless of changes of its p-value and/or coefficient in the next analysis. In the final model, we took into account a significance value of *p* < 0.05.

We decided to consider two-years time lag between predictors and outcome, to allow a reasonable period of time before exploring the effects of the different factors on stunting.

All the analyses we carried out took into account the survey sample design, including weights, clusters and strata. All analyses were conducted with Stata 13.1 (Stata Corp., College Station, TX).

## Results

### Time trends

**Fig. 2:**
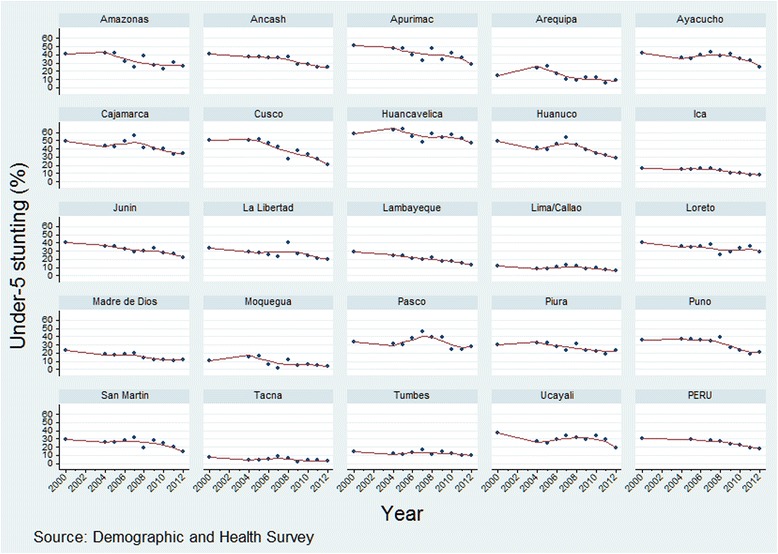
Departmental time trends of under-five stunting prevalence. Peru: 2000–2012

Stunting prevalence followed a decreasing trend in all departments, with different slopes. In general, the pace of reduction was more evident from 2007–2008 onwards (Fig 2).

Detailed time trends for the variables of the different dimensions of the conceptual framework are shown in Additional file 1. Most departments show improvement of social determinants, out-of-health sector, within health sector, and interventions coverage variables, as well as improvement of impact indicators, although at differing pace.

**Fig. 3:**
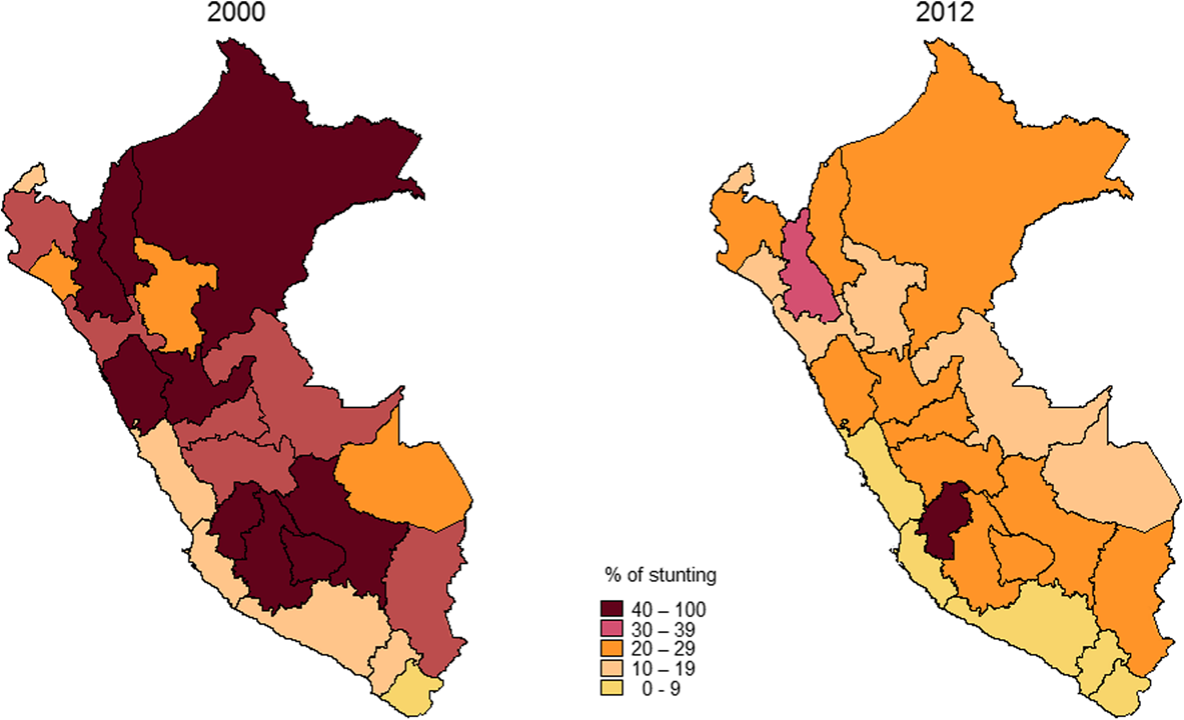
Under-five stunting prevalence by department, Peru: 2000 and 2012

When contrasting stunting prevalence between baseline year and most recent year, 15 departments had 30% or more under-five stunted children in 2000, compared with only 2 departments with such prevalence in 2012 (Fig 3).

Table 1 shows the ranking of departments by annual change of stunting prevalence in terms of beta coefficient. The departments with the highest annual reduction were Cusco (−2.31%), Amazonas (−1.57%), Puno (−1.54%), Huanuco (−1.52%), and Ancash (−1.44). Conversely, those with the lowest reduction were Ica (−0.67%), Ucayali (−0.64%), Tumbes (−0.45%), Lima (−0.37%), and Tacna (−0.31%).

**Table 1:** Ranking of departments by average annual absolute reduction of stunting prevalence (in percentage points)

	Beta	SE
1. Cusco	−2.31	0.52
2. Amazonas	−1.57	0.51
3. Puno	−1.54	0.46
4. Huanuco	−1.52	0.56
5. Ancash	−1.44	0.25
6. Lambayeque	−1.33	0.10
7. Apurimac	−1.33	0.52
8. Junin	−1.29	0.20
9. San Martin	−1.21	0.52
10. Cajamarca	−1.14	0.51
11. Madre de Dios	−1.13	0.16
12. Pasco	−0.96	0.79
13. Huancavelica	−0.93	0.44
14. Ayacucho	−0.92	0.41
15. Moquegua	−0.89	0.37
16. Loreto	−0.87	0.41
17. Arequipa	−0.81	0.46
18. Piura	−0.78	0.37
19. La Libertad	−0.75	0.54
20. Ica	−0.67	0.22
21. Ucayali	−0.64	0.58
22. Tumbes	−0.45	0.22
23. Lima	−0.37	0.21
24. Tacna	−0.31	0.17

*AAAR*: average absolute annual reduction; *SE*: standard error

Peru: DHS, 2000–2012

Similar rankings according to the annual change of different predictor variables of the conceptual framework are shown in Additional files 2, 3 and 4. The departments that show the highest reductions in stunting are not necessarily those that improved most their social determinants or their coverage of RMNCH interventions potentially influencing stunting.

### Ecological multilevel mixed effects analyses

The results were consistent when using either regression-based or tree-based imputations. For the sake of brevity, we only present in this report the results obtained by using the dataset that included the regression-based imputed values for the specified variables.

#### Time-adjusted linear multilevel models for under-five stunting prevalence

Time-adjusted coefficients showed that poverty line, at least one unmet basic need, urbanization, and women’s schooling influenced significantly the annual stunting reduction over the study period, with significant variation across departments (Table 2).

**Table 2:** Multilevel linear models for stunting prevalence (2 years time-lag)^a^

Dimension of the predictor variables	Predictor Variables	Time-adjusted regression coefficient	95% CI	*p*	Time and confounder-adjusted^a^ regression coefficient	95% CI	*p*
	Time (year)	−1.728	−2.056 to −1.399	0.000	−0.234	−0.862 to 0.395	0.466
Box A: Social determinants	log GDP per capita (USD/person)	−10.712	−17.174 to −4.250	0.001	-	-	-
	Unmet basic needs (at least 1) % of people	0.187	0.070 to 0.305	0.002	0.209	0.108 to 0.310	0.000
	Poverty line (% of people below)	0.416	0.302 to 0.529	0.000	0.196	0.073 to 0.319	0.002
	Gini coefficient for income	0.050	−0.204 to 0.305	0.699	-	-	-
	Urbanization (% of urban population)	−0.518	−0.626 to −0.409	0.000	−0.220	−0.374 to −0.066	0.005
Box B: Non-health sector variables	Years of schooling of women (median)	−2.839	−3.662 to −2.017	0.000	−0.928	−1.938 to 0.082	0.072
	Improved water source (% of households)	−0.005	−0.097 to 0.088	0.921	-	-	-
	Total fertility rate	1.241	−0.849 to 3.332	0.245	-	-	-
	Cash Transfer Programme Coverage (No. fam/1000 rural pop)	0.001	−0.033 to 0.036	0.944	-	-	-
Box C: Health sector variables	SIS utilization (Number of attendance/u5 pop)	−0.585	−1.528 to 0.359	0.224	-	-	-
	Per capita expenditure on child health activities (USD/u5 pop)	0.004	−0.009 to 0.016	0.569	-	-	-
	Density of human resources for health (per 10,000 pop)	−0.147	−0.454 to 0.161	0.351	-	-	-
	Composite coverage index (CCI)	0.166	−0.020 to 0.352	0.080	0.277	0.095 to 0.458	0.003

^a^Units of analyses are 168 (24 departments × 7 years). Variables in each group are adjusted for all other variables in the same group or above

#### Time- and confounding-adjusted linear multilevel models for under-five stunting prevalence

Similarly, when we controlled for time and confounding, social determinants (particularly percentage of families with at least one unmet basic need, percentage of families living under the poverty line, urbanization, and women’s schooling) seemed to be the most important factors influencing annual stunting reduction at departmental level (Table 2), with the random effects component of the model showing significant variation between departments. Composite coverage index showed a significant effect, but not in the expected direction. Similarly, when we controlled for time and confounding, social determinants (particularly percentage of families with at least one unmet basic need, percentage of families living under the poverty line, urbanization, and women’s schooling) seemed to be the most important factors influencing annual stunting reduction at departmental level (Table 2), with significant variation between departments.

## Discussion

Our results show that social determinants were more important factors influencing the annual reduction of under-five stunting in Peru than coverage of specific RMNCH interventions. This is in line with previous studies performed at national level in Peru and in other developing countries [4, 8, 25–27]. Of note, the departments where stunting prevalence was higher than 30% in 2000 and showed the greatest reduction by 2012 are all located in the Andean and the Amazon regions of the country. These are the regions that also show the highest rates of poverty and rural population in 2000, which were also reduced significantly during the study period. This highlights that the district level reduction of stunting in Peru followed a progressive pattern, with a clear reduction of the equity gaps between the richest and the poorest segments of the population and between urban and rural areas [11], which is a remarkable achievement.

Interestingly, GDP per capita showed a significant correlation in the time-adjusted analysis, but this effect disappeared when confounding factors were included ever since the first step of our regressions. Of note, Gini coefficient was not significant in our analyses. Vollmer et al. obtained similar results in a DHS-based study of 36 low-income and middle-income countries, where stunting was one was the outcome variables [28]. It seems that for economic growth to result in a measurable impact on stunting, various mediating factors need to play an enabling role, namely wealth distribution, poverty reduction, female education, improved access to safe water and sanitation, improved coverage and quality of maternal and child health interventions, as well as increased consumption of nutritious and safe food [28–30]. In Peru, there is evidence that anti-poverty interventions such as the conditional cash transfer (JUNTOS) programme might impact on stunting through improvement of food consumption by children [31], besides other factors, although its effect on nutrition may still need additional time, as our study did not reveal a positive impact of JUNTOS on stunting.

Mejía Acosta et al. tried recently to unveil the factors behind the remarkable reduction of child stunting in Peru, by using a veto players approach combined with quantitative information on time trends [8]. They emphasize that a comprehensive explanation of this successful story should take into account economic growth, poverty reduction, increased women’s education, reduced fertility rates, improved access to basic sanitary facilities, and urbanization, but also the concurrent role of widely concerted enabling policy and system factors, leading to increased equity and efficiency of health and other sectors able to provide essential services that can ensure a safe pregnancy, delivery and infancy.

Our district level results, showing that stunting reduction seemed to be more responsive to improvement of social determinants than to better coverage of specific RMNCH interventions, need to be interpreted within the wider policy framework that characterized Peru around the study period. During the 2000s political consensus, national and regional agreements were established with the participation of political parties, the civil society and other stakeholders [32–35]. They agreed on the need to prioritize multisectoral anti-poverty policies and to implement specific programmes aimed at improving maternal and child health. More specifically, specific goals for reduction of stunting were agreed on, which were translated into the implementation of specific crosscutting interventions such as reduction of poverty through quick introduction and scaling up of the conditional cash transfer programme JUNTOS in 2005 [36], through improved access to improved water and sanitation and women’s empowerment [36, 37], and through the extension of subsidized and semi-subsidized health provision through the health insurance system (SIS) to reach the poorest segments of the population. More recently, the Ministry of Economy and Finance led the implementation of multisectoral nutritional and maternal-neonatal programmes relying on the results-based budgeting approach, with the aim to increase the efficiency and the effectiveness of expenditure at central and local levels, and to further reduce stunting and maternal and neonatal mortality [38–40]. Under these programmes, the budget is allocated based on the performance reached by each region, in terms of coverage and impact indicators [38–40].

Conditional cash transfer and health insurance system (SIS) showed a significant association with stunting prevalence. That is, the higher the coverage of those interventions, the higher the stunting prevalence. This may be reflecting the fact that both programmes have been reaching effectively the poorest segments of the population, although further analyses may be warranted to reach a definitive conclusion. Similarly, the composite coverage index showed a significant and positive correlation with stunting, which is in the opposite direction to the expected. As this is a composite indicator and should overcome the limitations posed by insufficient sample size, we would have anticipated a significant and negative association instead, thus we do not have a clear explanation for this rather unexpected result.

There are potential limitations in our study that need to be acknowledged. Collinearity could have been an issue, although we tried to avoid this by excluding variables with similar construct, such as poverty and extreme poverty indicators. Sample size constraints could also explain the lack of influence of some variables on stunting reduction, particularly those related to specific RMNCH interventions such as coverage of vaccines and coverage of care-seeking for pneumonia. Possible relevant interactions between variables cannot be ruled out, although they are not easy to measure. Also, variables measuring quality of care could be better suited to identify actual effects on health in mixed effects regression models such as the one we used. Finally, there is the possibility that our model may need additional refinement to better capture the complex network of interacting variables of different dimensions that may influence stunting in under-five children.

We think that future studies at sub-national level should consider using a combination of quantitative and qualitative approaches that take into account the complex role of social, political, policy, financial, and technical aspects underlying the implementation of specific nutritional and proximal interventions more directly related to stunting. Such an approach will need significant improvement of availability, completeness and accuracy of departmental level data collected on a routine basis and through periodic surveys. Specifically, we suggest that DHS and other relevant surveys should include variables measuring quality of services provided rather than merely measuring quantity, such as quality of antenatal care visits and quality of birth attendance. It is encouraging that this is already happening in Peru, where DHS has incorporated recently quality indicators for interventions such as obstetric functionality and solving capacity of health facilities, quality of antenatal care visits, and quality of healthy child monitoring visits [41, 42].

In addition, further strengthening of regional and local political, managerial and technical capabilities will be needed, to facilitate both effective crosscutting implementation of interventions and a fully functional monitoring and evaluation system, so as to ensure a sustained stunting reduction, with continued focus on the poorest areas of the country.

## Conclusion

After a long period with little change, Peru was able to reduce dramatically its under-five stunting prevalence at national and departmental level, thanks to the reduction of poverty and to a sustained and equitable implementation of multisectoral interventions. This success story may offer useful lessons to other countries trying to improve the nutrition of their children.

## Additional files

Additional file 1: Departmental time trends of main variables of the conceptual framework [7, 15–18]. (DOCX 1619 kb)
Additional file 2: Ranking of departments by annual change of social determinants and out-of-health sector factors [7, 15–18]. (DOCX 126 kb)
Additional file 3: Ranking of departments by annual variation of health sector factors [16, 17]. (DOCX 67 kb)
Additional file 4: Ranking of departments by annual variation of coverage of RMNCH interventions [7]. (DOCX 108 kb)
Additional file 5: Stunting__Resubmitted_BMCP_December2016 [7, 15–18]. (XLS 637 kb)

## Abbreviations

DHS: Demographic and health surveys
GDP: Gross domestic product
JUNTOS: Peruvian conditional cash transfer programme
RMNCH: Reproductive, maternal, neonatal and child health
SIS: Peruvian comprehensive health insurance system
