# Supplementary material for: Factors behind the success story of under-five stunting in Peru: a district ecological multilevel analysis
Source: BMC Pediatr. 2017 Jan 19;17:29. doi: 10.1186/s12887-017-0790-3 (PMC5248498; doi:10.1186/s12887-017-0790-3)
Supplement: Additional file 1: — Departmental time trends of main variables of the conceptual framework [7, 15–18]. (DOCX 1619 kb) [file 12887_2017_790_MOESM1_ESM.docx]

**Additional file 1. Departmental time trends of main variables of the conceptual framework
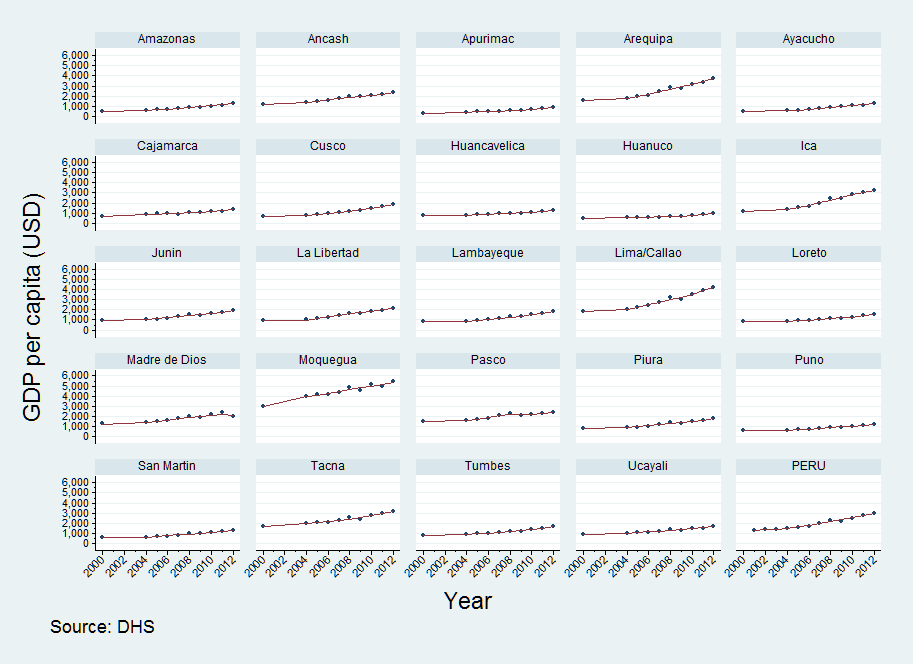
**

**
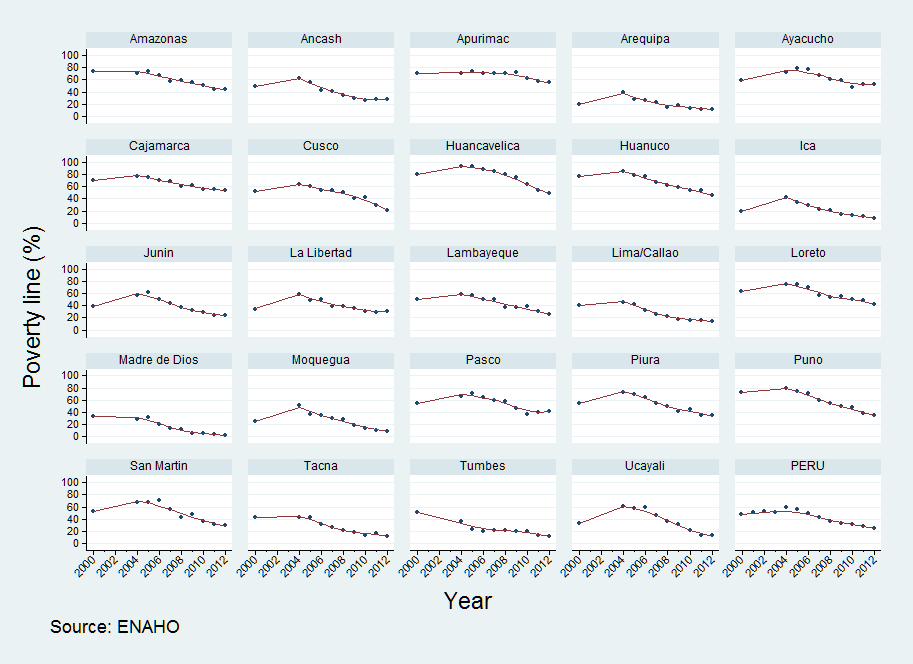
**

**
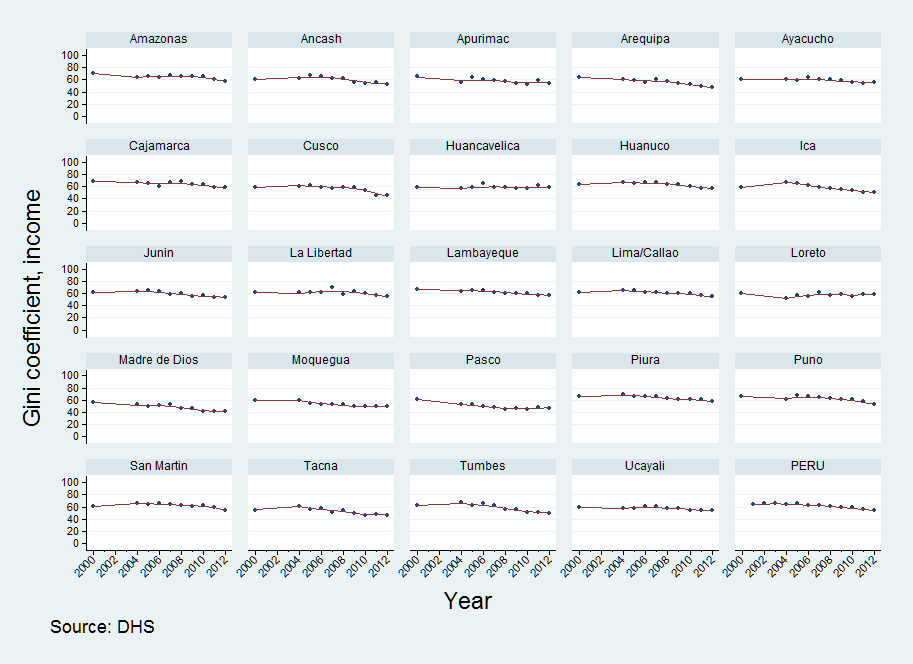
**

**
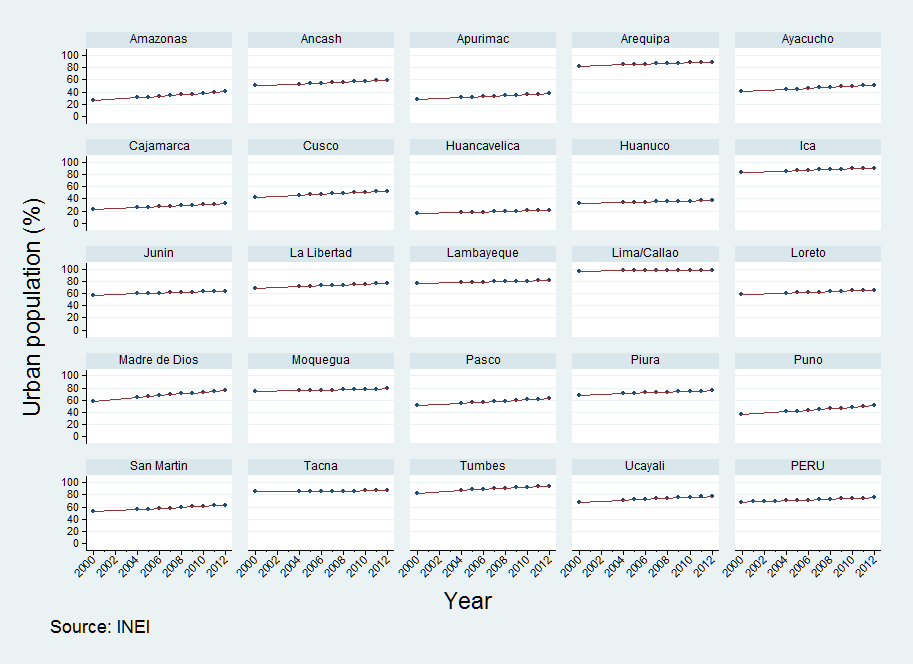
**

**
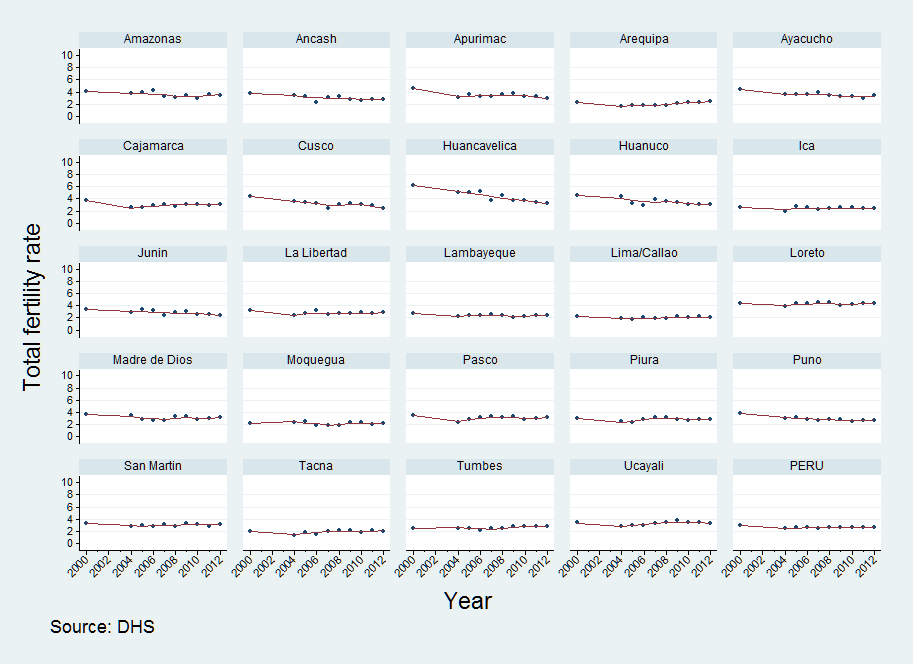
**

**
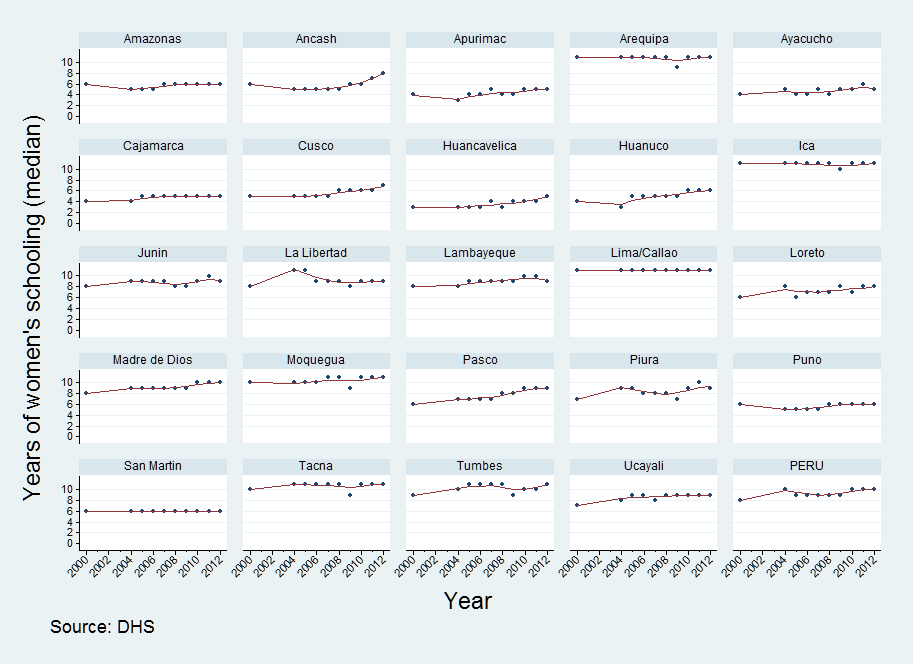
**

**
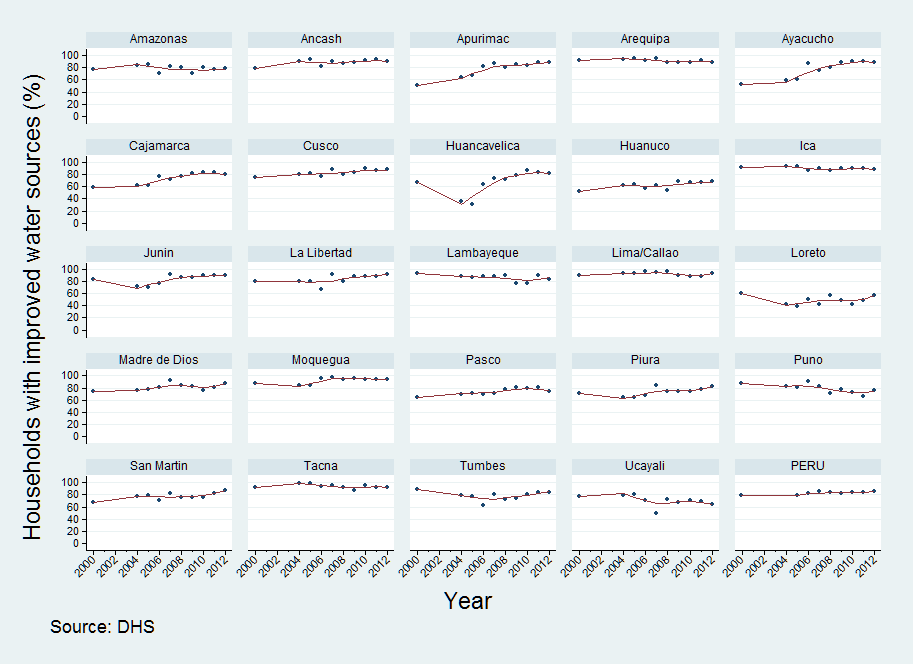
**

**
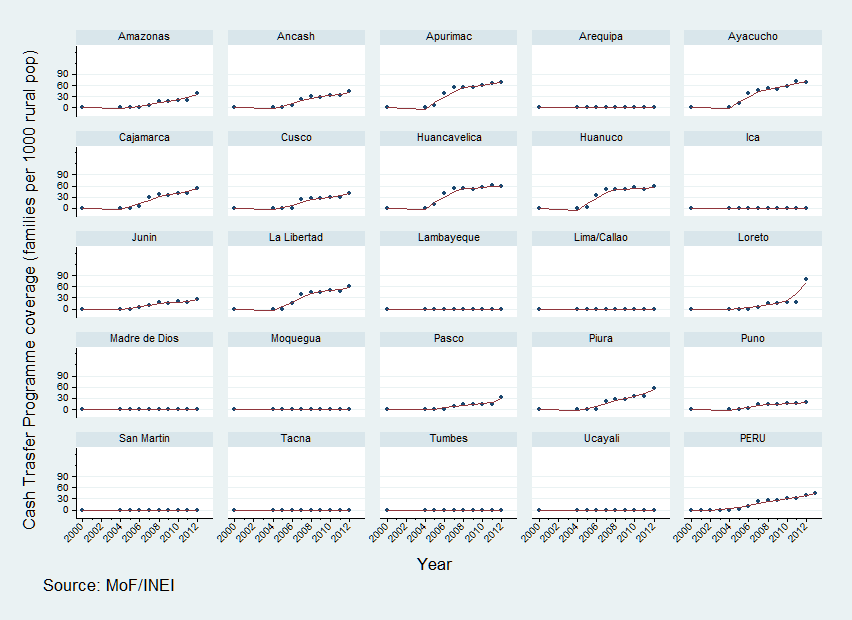
**

**
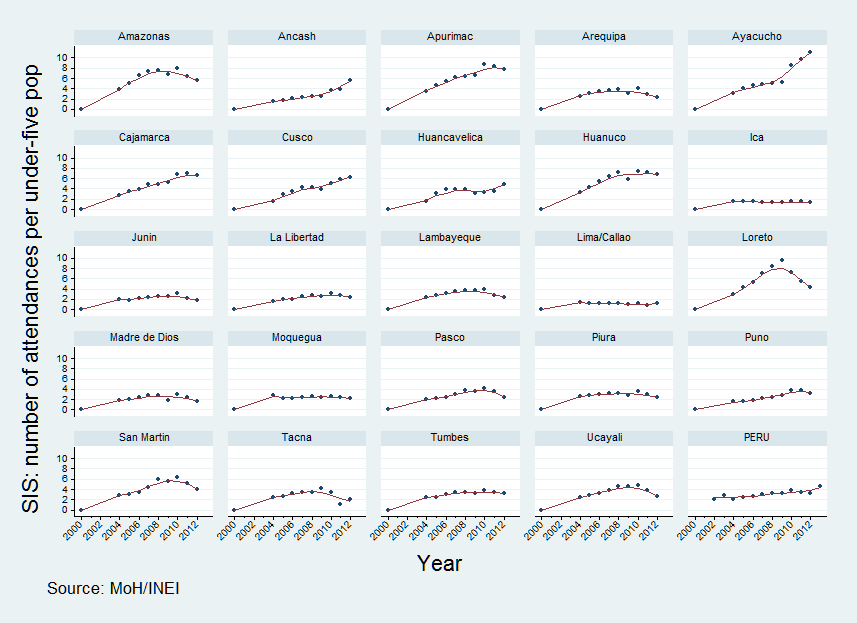
**

**
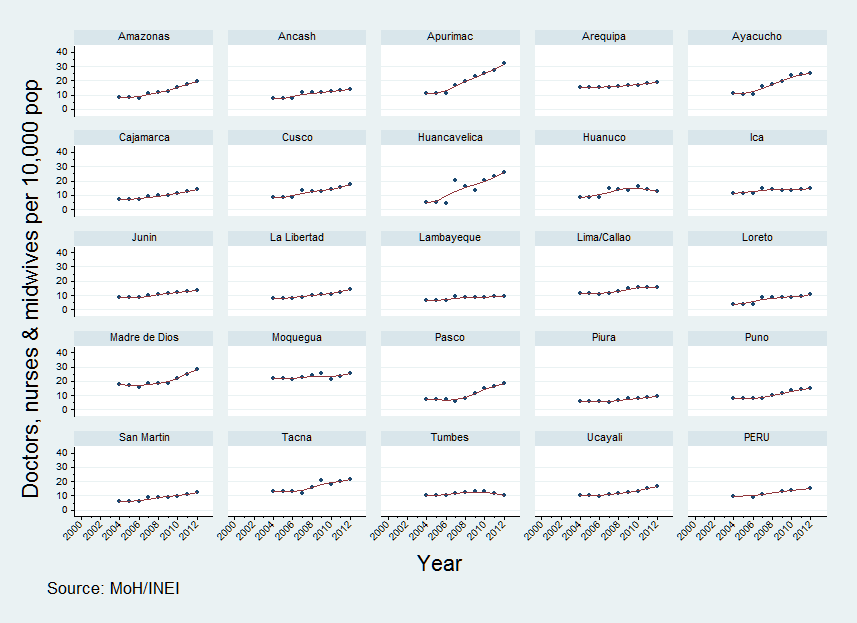
**

**
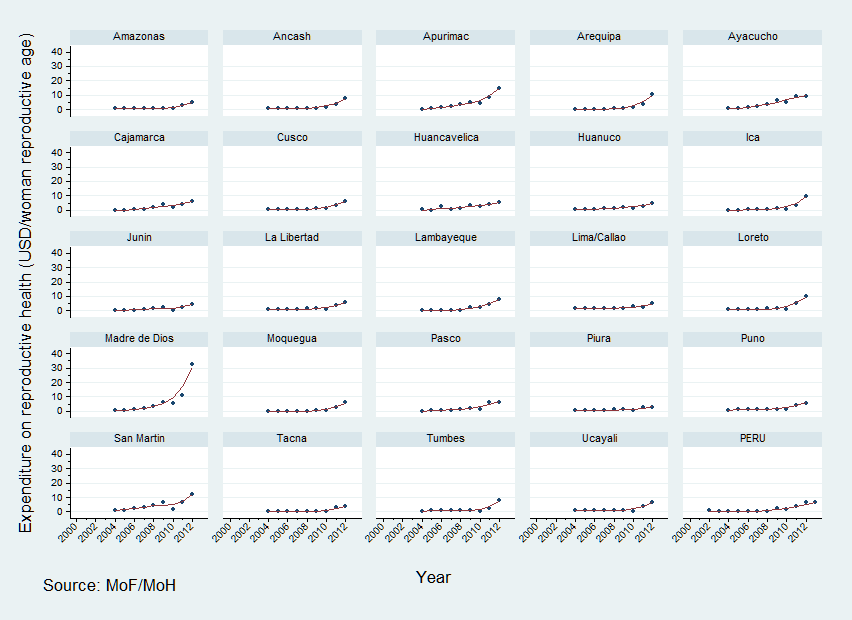
**

**
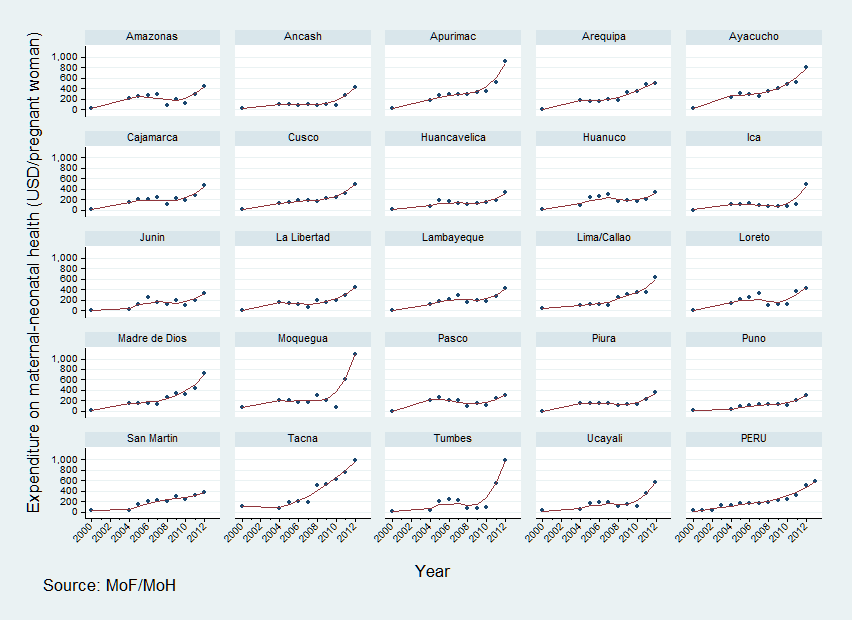
**

**
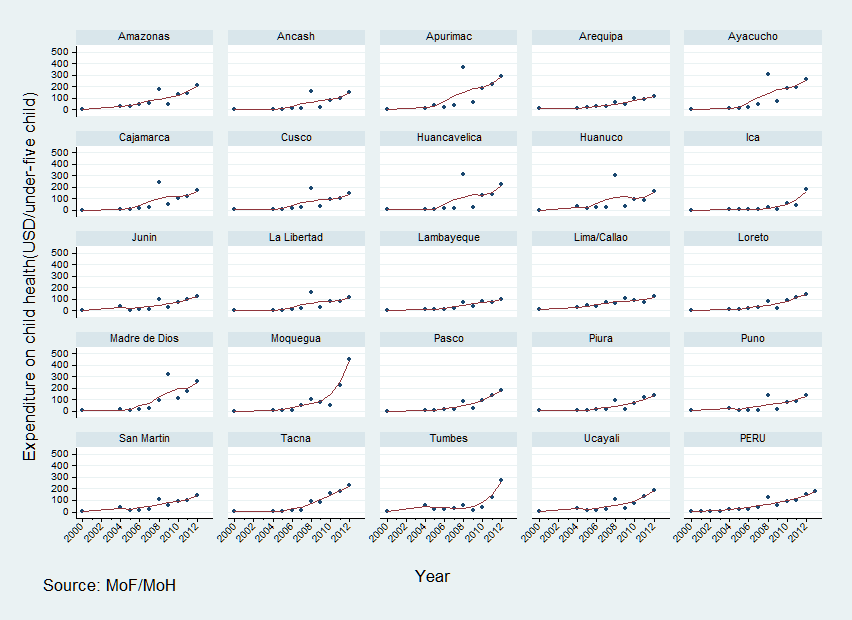
**

**
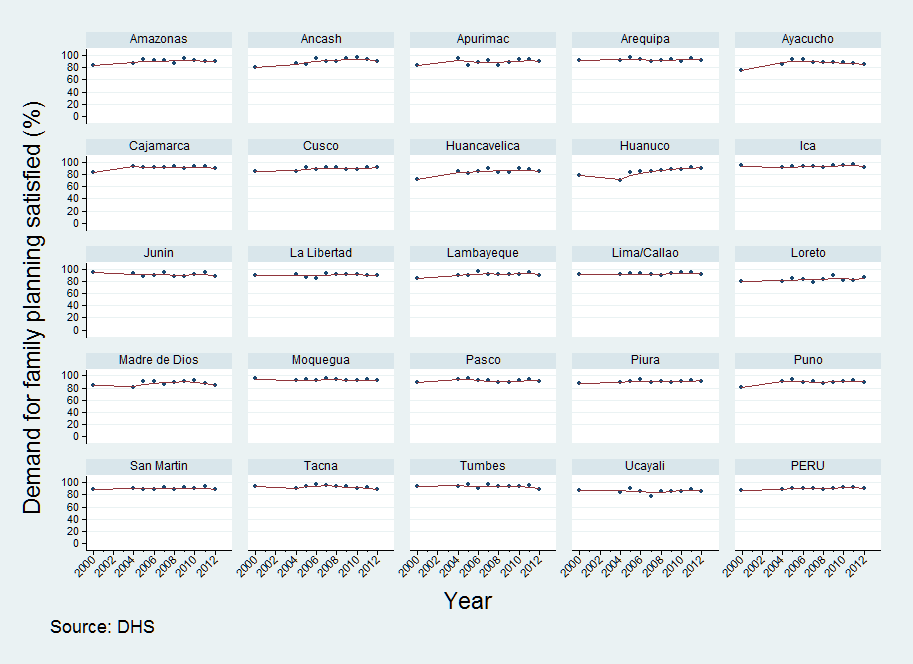
**

**
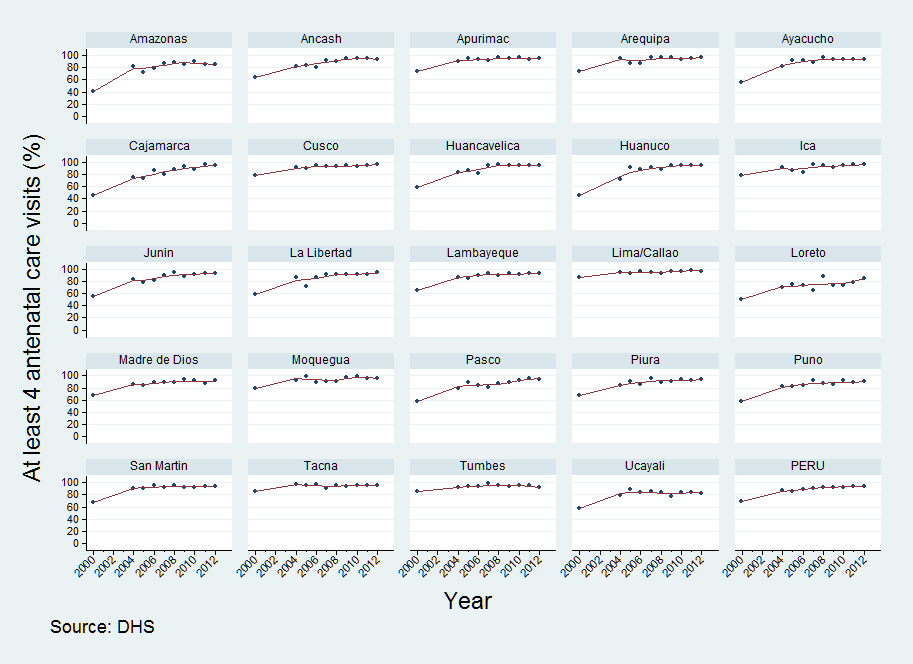
**

**
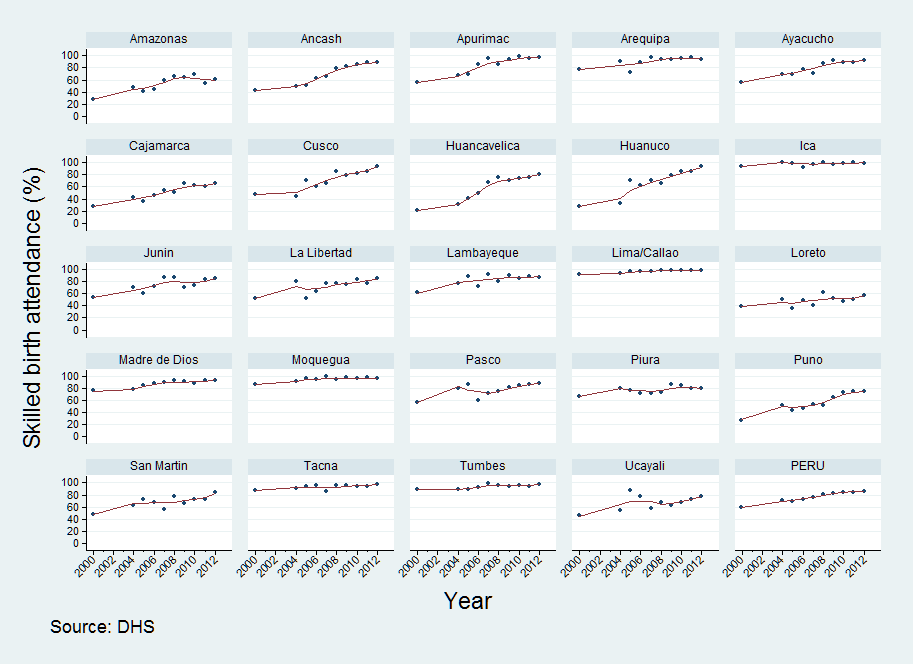
**

**
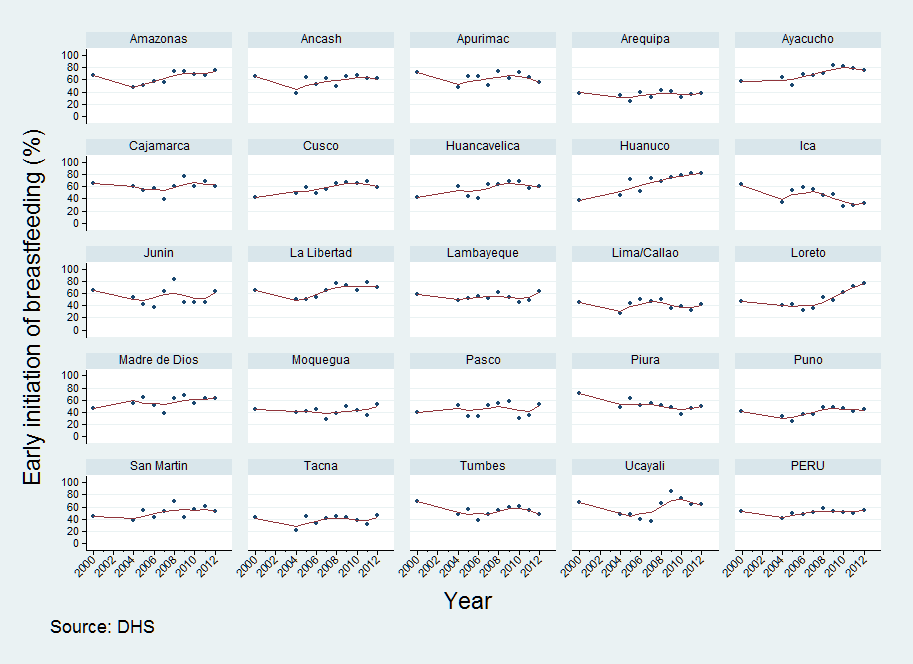
**

**
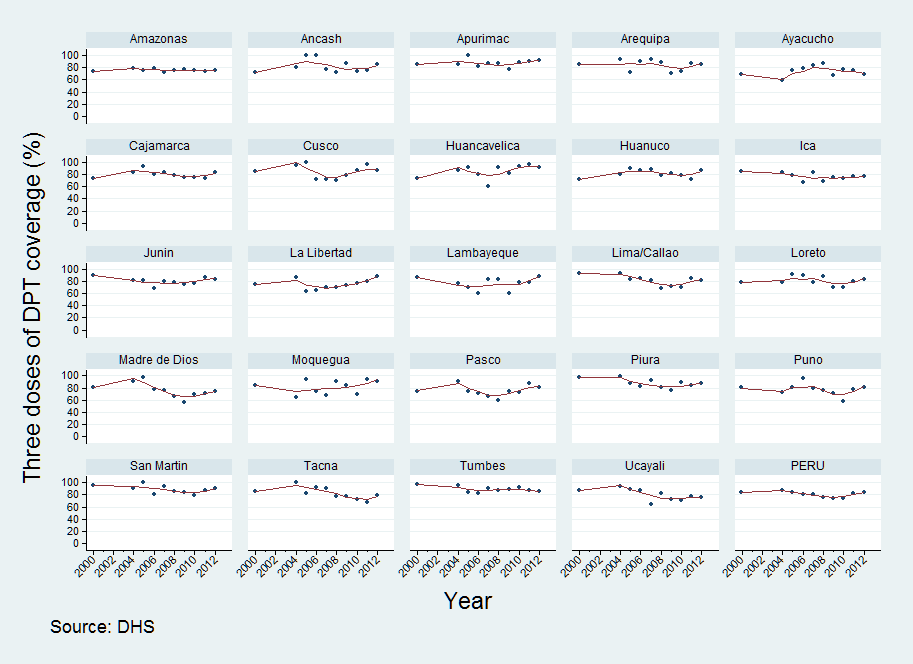
**

**
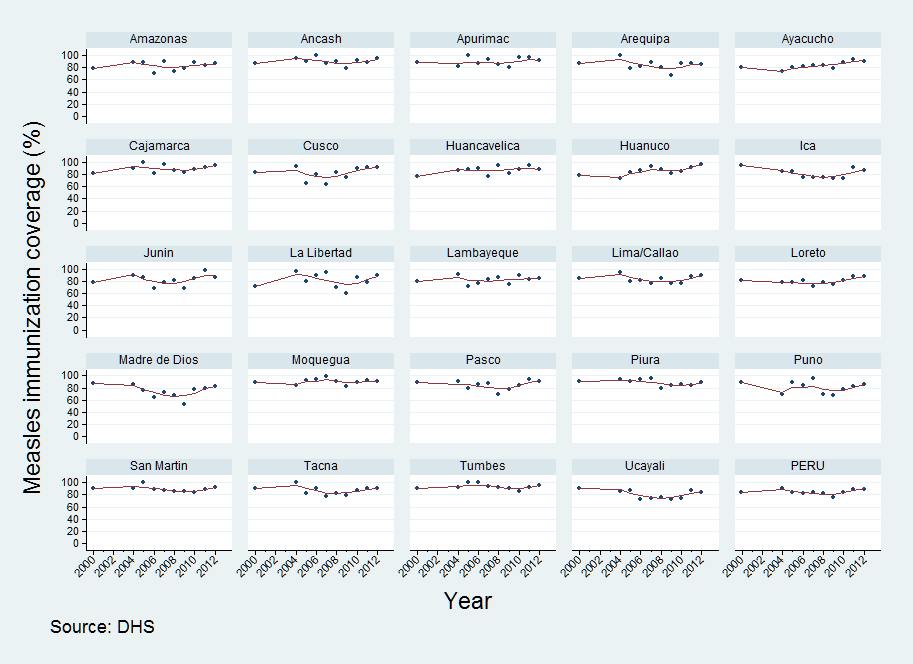
**

**
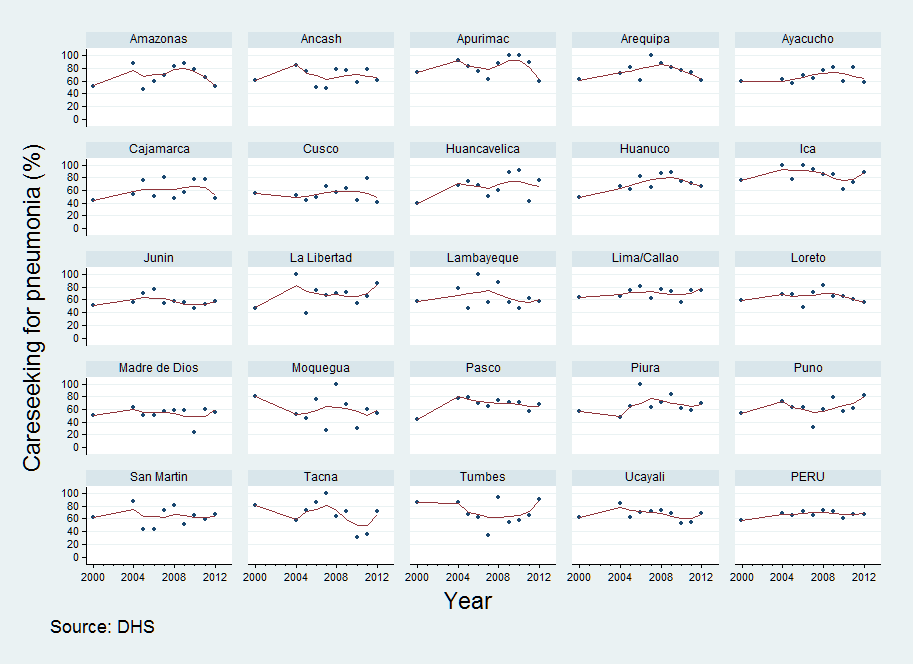
**

**
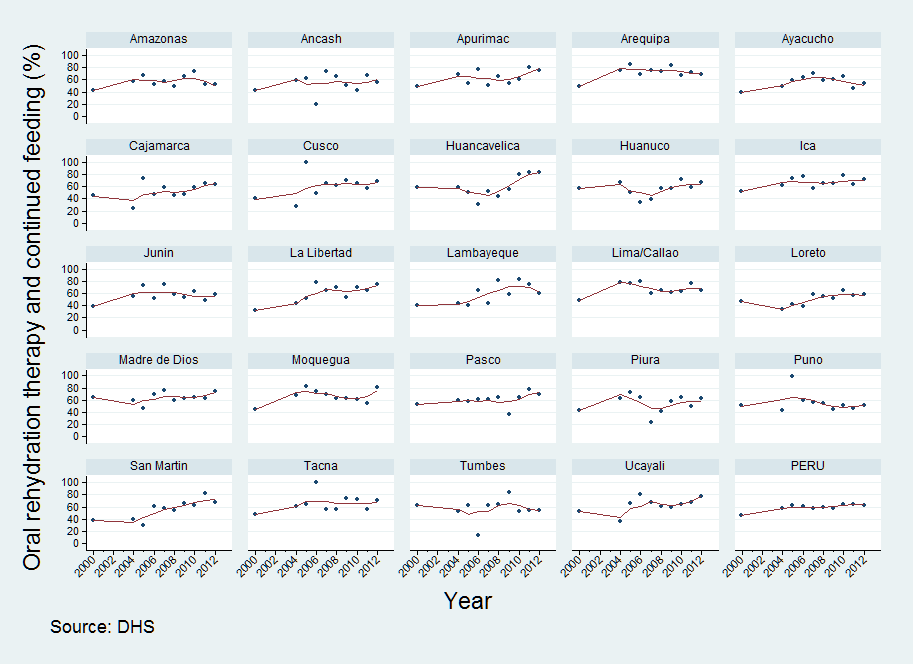
**

**
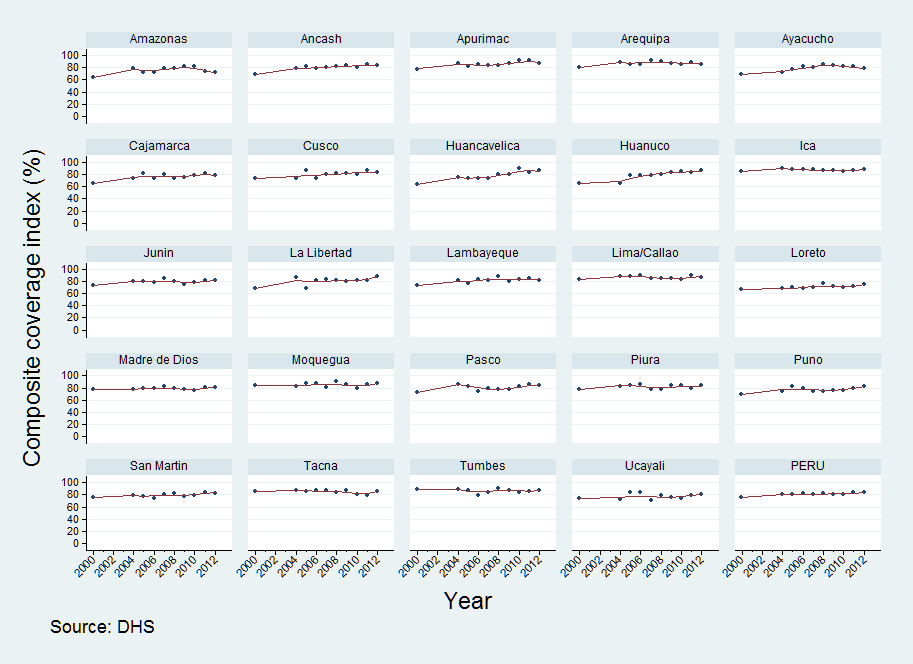
**

**
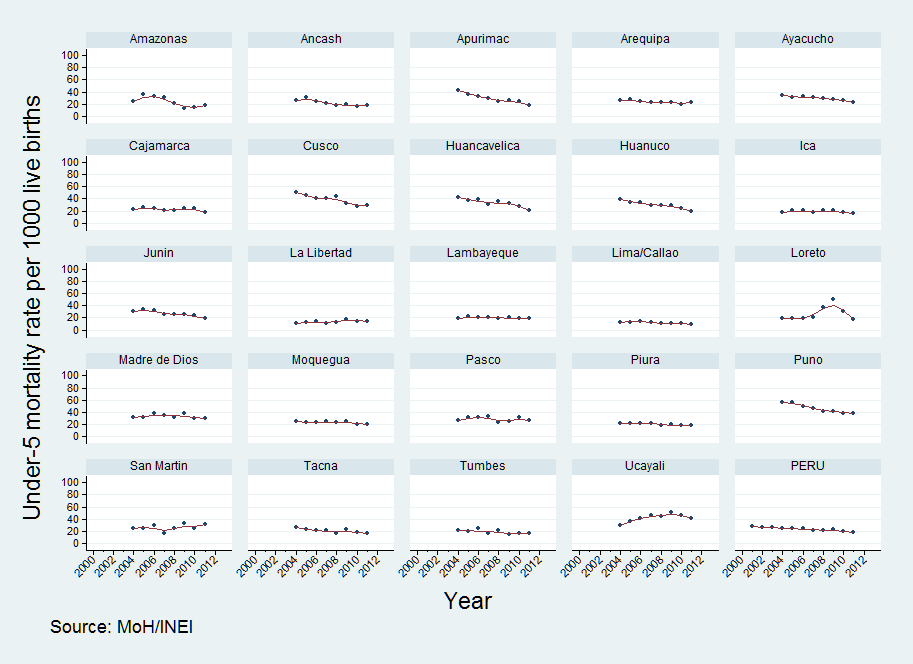
**

**
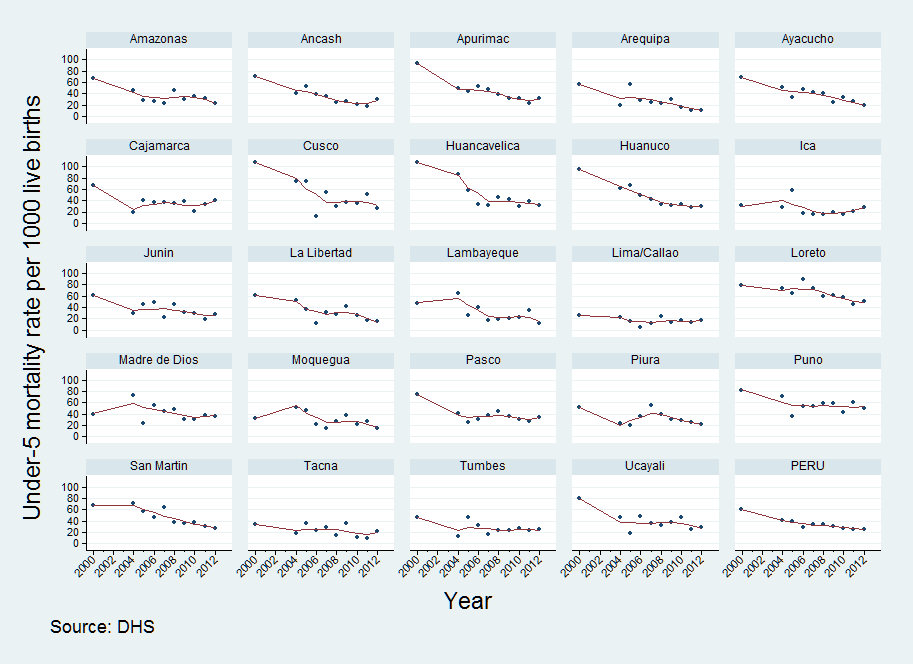
**

**
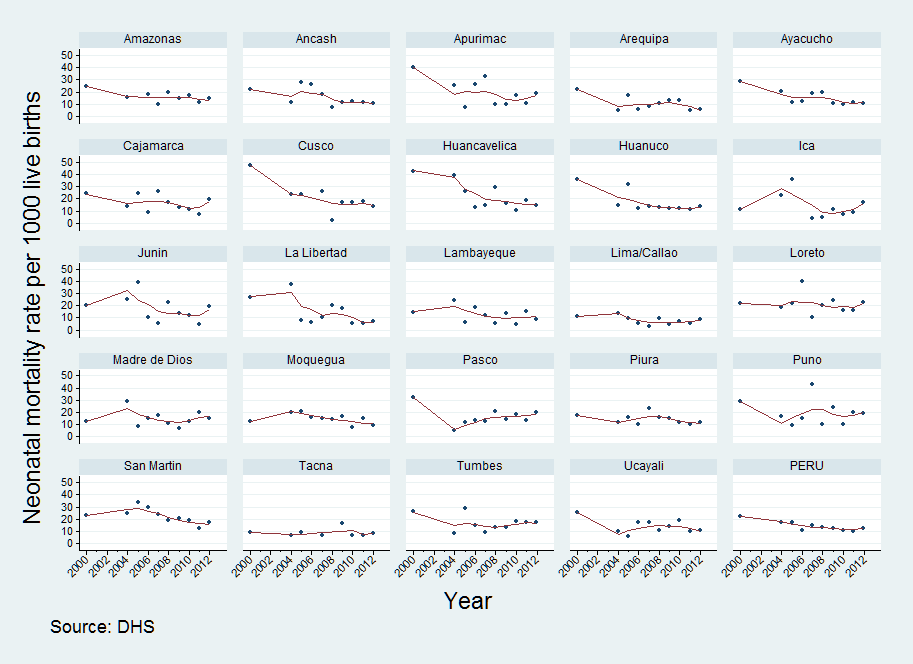
**

**
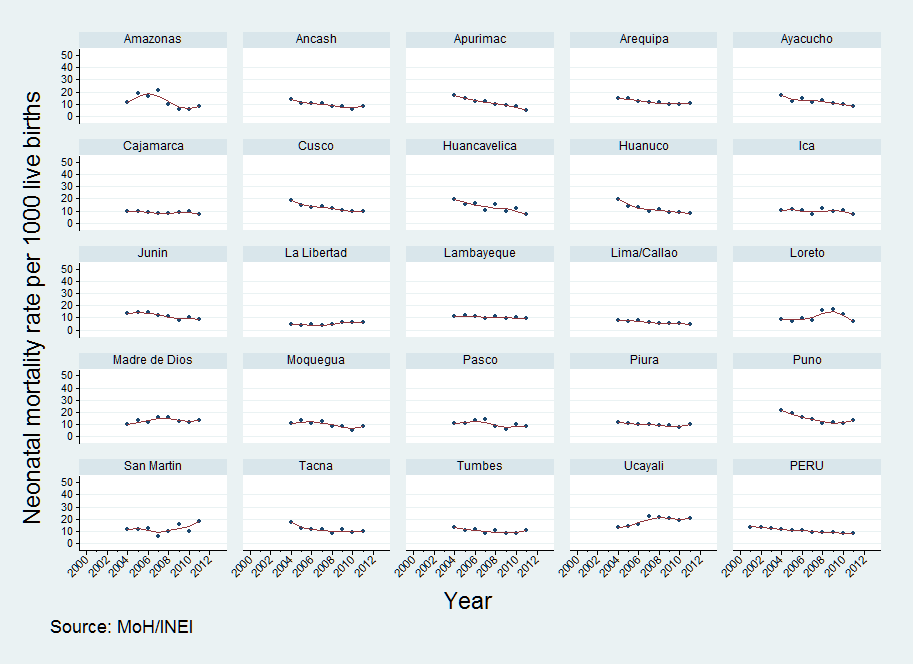
**

**DHS: Demographic and Health Survey. ENAHO: Notional Household Survey. INEI: Institute of Statistics and Computing. MoF: Ministry of Economy and Finance. MOH: Ministry of Health.**
