# Supplementary material for: Factors behind the success story of under-five stunting in Peru: a district ecological multilevel analysis
Source: BMC Pediatr. 2017 Jan 19;17:29. doi: 10.1186/s12887-017-0790-3 (PMC5248498; doi:10.1186/s12887-017-0790-3)
Supplement: Additional file 2: — Ranking of departments by annual change of social determinants and out-of-health sector factors [7, 15–18]. (DOCX 126 kb) [file 12887_2017_790_MOESM2_ESM.docx]

| **Additional file 2. Ranking of departments by annual change of social determinants and out-of-health sector factors.** | | | | | | | | | | | | | | |  |  |  |  |
| --- | --- | --- | --- | --- | --- | --- | --- | --- | --- | --- | --- | --- | --- | --- | --- | --- | --- | --- |
| **Department** | **GDP per capita (USD)** | | **Gini coefficient income** | | **Poverty line** | | **At least one unmet basic need** | | **Urbanization** | | **Women's schooling** | | **Fertility rate** | | **Improved water source** | | **Rural families beneficiaries of JUNTOS** | |
|  | **Beta** | **SE** | **Beta** | **SE** | **Beta** | **SE** | **Beta** | **SE** | Beta | SE | **Beta** | **SE** | Beta | SE | Beta | SE | **Beta** | **SE** |
| **Amazonas** | 65.16 | 7.81 | -0.75 | 0.26 | -2.91 | 0.43 | -1.71 | 0.28 | 1.27 | 0.02 | 0.06 | 0.04 | -0.07 | 0.03 | -0.30 | 0.46 | 3.09 | 0.62 |
| **Ancash** | 102.87 | 7.54 | -0.88 | 0.35 | -2.87 | 0.69 | -2.16 | 0.33 | 0.75 | 0.00 | 0.17 | 0.08 | -0.08 | 0.03 | 0.90 | 0.36 | 4.12 | 0.66 |
| **Apurimac** | 43.07 | 5.33 | -0.81 | 0.31 | -1.28 | 0.47 | -2.51 | 0.45 | 0.85 | 0.01 | 0.12 | 0.05 | -0.09 | 0.03 | 3.21 | 0.51 | 6.95 | 1.13 |
| **Arequipa** | 192.47 | 19.80 | -1.31 | 0.21 | -1.55 | 0.69 | -0.80 | 0.15 | 0.52 | 0.01 | -0.03 | 0.06 | 0.03 | 0.03 | -0.34 | 0.24 | 0.00 | 0.00 |
| **Ayacucho** | 68.47 | 8.04 | -0.52 | 0.20 | -1.63 | 0.87 | -2.55 | 0.52 | 0.95 | 0.00 | 0.12 | 0.05 | -0.10 | 0.02 | 3.71 | 0.66 | 6.87 | 0.90 |
| **Cajamarca** | 47.71 | 4.38 | -0.74 | 0.29 | -1.99 | 0.45 | -2.27 | 0.41 | 0.78 | 0.01 | 0.09 | 0.03 | -0.03 | 0.03 | 2.35 | 0.39 | 5.20 | 0.85 |
| **Cusco** | 106.52 | 12.66 | -1.13 | 0.40 | -2.86 | 0.83 | -3.35 | 0.22 | 0.91 | 0.00 | 0.16 | 0.04 | -0.13 | 0.03 | 1.10 | 0.31 | 3.89 | 0.73 |
| **Huancavelica** | 44.14 | 5.65 | -0.01 | 0.27 | -3.21 | 1.01 | -4.98 | 0.47 | 0.52 | 0.01 | 0.15 | 0.04 | -0.26 | 0.03 | 3.27 | 1.47 | 6.12 | 1.14 |
| **Huanuco** | 36.44 | 4.75 | -0.71 | 0.27 | -3.14 | 0.62 | -3.67 | 0.68 | 0.45 | 0.00 | 0.21 | 0.05 | -0.12 | 0.04 | 1.21 | 0.41 | 5.95 | 1.17 |
| **Ica** | 196.33 | 20.63 | -1.16 | 0.37 | -2.05 | 0.82 | -0.93 | 0.43 | 0.67 | 0.02 | -0.02 | 0.03 | 0.00 | 0.02 | -0.29 | 0.23 | 0.00 | 0.00 |
| **Junin** | 84.56 | 9.96 | -0.96 | 0.26 | -2.43 | 0.97 | -1.65 | 0.17 | 0.55 | 0.00 | 0.07 | 0.06 | -0.08 | 0.03 | 1.30 | 0.63 | 2.43 | 0.37 |
| **La Libertad** | 113.32 | 11.22 | -0.44 | 0.36 | -1.46 | 0.81 | -1.65 | 0.16 | 0.70 | 0.01 | -0.05 | 0.10 | -0.02 | 0.03 | 1.15 | 0.63 | 5.93 | 0.97 |
| **Lambayeque** | 86.33 | 11.57 | -0.87 | 0.15 | -2.59 | 0.62 | -1.29 | 0.31 | 0.43 | 0.01 | 0.15 | 0.04 | -0.02 | 0.01 | -0.80 | 0.43 | 0.00 | 0.00 |
| **Lima** | 210.93 | 24.67 | -0.76 | 0.19 | -2.96 | 0.55 | -0.84 | 0.11 | 0.11 | 0.00 | 0.00 | 0.00 | 0.01 | 0.01 | -0.10 | 0.29 | 0.00 | 0.00 |
| **Loreto** | 60.88 | 7.40 | 0.02 | 0.29 | -2.49 | 0.70 | 0.35 | 0.36 | 0.64 | 0.00 | 0.14 | 0.06 | 0.01 | 0.02 | -0.09 | 0.70 | 4.61 | 1.69 |
| **Madre de Dios** | 91.18 | 13.73 | -1.38 | 0.21 | -3.13 | 0.39 | -1.99 | 0.17 | 1.42 | 0.02 | 0.16 | 0.03 | -0.04 | 0.03 | 0.84 | 0.46 | 0.00 | 0.00 |
| **Moquegua** | 189.52 | 14.58 | -1.02 | 0.17 | -2.48 | 0.92 | -1.77 | 0.22 | 0.40 | 0.00 | 0.09 | 0.06 | -0.01 | 0.03 | 0.94 | 0.39 | 0.00 | 0.00 |
| **Pasco** | 83.32 | 10.81 | -1.27 | 0.24 | -2.28 | 0.87 | -1.92 | 0.22 | 0.90 | 0.01 | 0.28 | 0.03 | -0.02 | 0.03 | 1.26 | 0.29 | 2.36 | 0.54 |
| **Piura** | 90.64 | 9.33 | -0.87 | 0.18 | -2.79 | 0.91 | -2.04 | 0.21 | 0.63 | 0.01 | 0.13 | 0.08 | 0.01 | 0.03 | 1.15 | 0.53 | 4.81 | 0.88 |
| **Puno** | 55.21 | 6.02 | -0.86 | 0.28 | -3.79 | 0.68 | -1.85 | 0.16 | 1.21 | 0.01 | 0.06 | 0.05 | -0.10 | 0.02 | -1.60 | 0.48 | 1.89 | 0.33 |
| **San Martin** | 62.64 | 6.50 | -0.43 | 0.29 | -3.02 | 1.06 | -1.59 | 0.10 | 0.89 | 0.01 | 0.00 | 0.00 | 0.00 | 0.02 | 1.09 | 0.40 | 0.00 | 0.00 |
| **Tacna** | 123.62 | 13.90 | -1.07 | 0.30 | -3.21 | 0.46 | -0.37 | 0.29 | 0.15 | 0.00 | 0.03 | 0.07 | 0.03 | 0.02 | -0.30 | 0.31 | 0.00 | 0.00 |
| **Tumbes** | 74.47 | 8.93 | -1.48 | 0.35 | -2.95 | 0.42 | -2.18 | 0.33 | 0.91 | 0.05 | 0.06 | 0.08 | 0.04 | 0.02 | -0.09 | 0.73 | 0.00 | 0.00 |
| **Ucayali** | 70.11 | 6.48 | -0.40 | 0.18 | -3.20 | 1.38 | 0.41 | 0.82 | 0.82 | 0.01 | 0.15 | 0.04 | 0.03 | 0.03 | -1.14 | 0.76 | 0.00 | 0.00 |
