# Supplementary material for: Factors behind the success story of under-five stunting in Peru: a district ecological multilevel analysis
Source: BMC Pediatr. 2017 Jan 19;17:29. doi: 10.1186/s12887-017-0790-3 (PMC5248498; doi:10.1186/s12887-017-0790-3)
Supplement: Additional file 3: — Ranking of departments by annual variation of health sector factors [16, 17]. (DOCX 67 kb) [file 12887_2017_790_MOESM3_ESM.docx]

| **Additional file 3. Ranking of departments by annual variation of health sector factors.** | | | | | | |
| --- | --- | --- | --- | --- | --- | --- |
| **Department** | **SIS attendances per under-five child** | | **Per capita expenditure on child health** | | **Doctors, nurses & midwives per 10,000 pop** | |
|  | **Beta** | **SE** | **Beta** | **SE** | Beta | SE |
| **Amazonas** | 0.50 | 0.15 | 16.14 | 4.18 | 1.23 | 0.13 |
| **Ancash** | 0.40 | 0.05 | 12.19 | 4.45 | 0.85 | 0.12 |
| **Apurimac** | 0.69 | 0.07 | 25.38 | 9.16 | 2.44 | 0.18 |
| **Arequipa** | 0.19 | 0.09 | 9.71 | 1.67 | 0.51 | 0.06 |
| **Ayacucho** | 0.87 | 0.10 | 23.75 | 7.52 | 1.96 | 0.15 |
| **Cajamarca** | 0.58 | 0.04 | 15.12 | 5.88 | 0.84 | 0.08 |
| **Cusco** | 0.52 | 0.04 | 12.08 | 4.77 | 1.22 | 0.13 |
| **Huancavelica** | 0.32 | 0.08 | 18.08 | 8.44 | 1.72 | 0.53 |
| **Huanuco** | 0.59 | 0.09 | 12.55 | 7.92 | 0.37 | 0.27 |
| **Ica** | 0.08 | 0.04 | 10.26 | 3.99 | 0.64 | 0.15 |
| **Junin** | 0.16 | 0.06 | 9.71 | 2.88 | 0.69 | 0.06 |
| **La Libertad** | 0.21 | 0.04 | 10.43 | 3.87 | 0.73 | 0.07 |
| **Lambayeque** | 0.20 | 0.09 | 8.61 | 1.75 | 0.40 | 0.11 |
| **Lima** | 0.05 | 0.03 | 8.79 | 1.42 | 0.88 | 0.13 |
| **Loreto** | 0.51 | 0.21 | 11.73 | 2.63 | 0.70 | 0.11 |
| **Madre de Dios** | 0.15 | 0.07 | 23.32 | 7.43 | 0.83 | 0.24 |
| **Moquegua** | 0.15 | 0.06 | 27.83 | 9.47 | 0.49 | 0.19 |
| **Pasco** | 0.27 | 0.07 | 14.42 | 3.58 | 1.17 | 0.23 |
| **Piura** | 0.18 | 0.07 | 11.03 | 3.00 | 0.52 | 0.08 |
| **Puno** | 0.31 | 0.03 | 10.35 | 3.87 | 0.67 | 0.11 |
| **San Martin** | 0.43 | 0.10 | 11.19 | 2.67 | 0.61 | 0.10 |
| **Tacna** | 0.13 | 0.11 | 20.20 | 4.06 | 1.29 | 0.21 |
| **Tumbes** | 0.26 | 0.06 | 14.09 | 6.11 | 0.07 | 0.10 |
| **Ucayali** | 0.29 | 0.10 | 13.35 | 3.85 | 0.74 | 0.12 |
