# Supplementary material for: Factors behind the success story of under-five stunting in Peru: a district ecological multilevel analysis
Source: BMC Pediatr. 2017 Jan 19;17:29. doi: 10.1186/s12887-017-0790-3 (PMC5248498; doi:10.1186/s12887-017-0790-3)
Supplement: Additional file 4: — Ranking of departments by annual variation of coverage of RMNCH interventions [7]. (DOCX 108 kb) [file 12887_2017_790_MOESM4_ESM.docx]

| **Additional file 4. Ranking of departments by annual variation of coverage of RMNCH interventions.** | | | | | | | | | | | | | | |
| --- | --- | --- | --- | --- | --- | --- | --- | --- | --- | --- | --- | --- | --- | --- |
| **Department** | **Family planning satisfied** | | **At least 4 antenatal care visits** | | **Infants with DPT vaccine** | | **Infants with measles vaccine** | | **Care-seeking for pneumonia** | | **Children with diarrhoea receiving ORT** | | **Composite coverage index (CCI)** | |
|  | **Beta** | **SE** | **Beta** | **SE** | **Beta** | **SE** | **Beta** | **SE** | **Beta** | **SE** | **Beta** | **SE** | **Beta** | **SE** |
| **Amazonas** | 0.54 | 0.28 | 3.26 | 0.86 | -0.03 | 0.22 | 0.31 | 0.66 | 0.84 | 1.52 | 0.80 | 0.87 | 0.80 | 0.47 |
| **Ancash** | 1.01 | 0.32 | 2.55 | 0.37 | -0.16 | 1.04 | -0.05 | 0.57 | 0.04 | 1.29 | 0.95 | 1.51 | 1.10 | 0.22 |
| **Apurimac** | 0.48 | 0.38 | 1.56 | 0.42 | 0.20 | 0.59 | 0.43 | 0.65 | 0.43 | 1.38 | 1.62 | 0.97 | 0.97 | 0.24 |
| **Arequipa** | 0.02 | 0.20 | 1.63 | 0.46 | -0.47 | 0.83 | -0.62 | 0.80 | 0.54 | 1.22 | 1.14 | 0.93 | 0.42 | 0.29 |
| **Ayacucho** | 0.47 | 0.45 | 2.70 | 0.66 | 0.55 | 0.77 | 1.23 | 0.39 | 0.95 | 0.86 | 0.92 | 0.87 | 1.05 | 0.32 |
| **Cajamarca** | 0.52 | 0.24 | 3.85 | 0.53 | -0.22 | 0.60 | 0.47 | 0.60 | 1.26 | 1.35 | 1.71 | 1.22 | 0.99 | 0.38 |
| **Cusco** | 0.40 | 0.20 | 1.24 | 0.29 | -0.16 | 1.05 | 0.69 | 0.97 | 0.41 | 1.10 | 1.97 | 1.76 | 0.90 | 0.37 |
| **Huancavelica** | 1.08 | 0.34 | 2.86 | 0.57 | 1.48 | 0.96 | 0.87 | 0.52 | 2.00 | 1.59 | 2.57 | 1.45 | 1.98 | 0.27 |
| **Huanuco** | 1.43 | 0.38 | 3.73 | 0.77 | 0.26 | 0.65 | 1.28 | 0.46 | 1.77 | 0.99 | 0.95 | 1.12 | 1.88 | 0.32 |
| **Ica** | 0.13 | 0.17 | 1.36 | 0.36 | -0.80 | 0.52 | -0.77 | 0.72 | -0.67 | 1.18 | 1.18 | 0.73 | 0.02 | 0.13 |
| **Junin** | -0.33 | 0.29 | 2.88 | 0.53 | -0.33 | 0.56 | 0.68 | 0.89 | -0.45 | 0.85 | 0.78 | 1.05 | 0.37 | 0.28 |
| **La Libertad** | 0.14 | 0.26 | 2.66 | 0.57 | 0.69 | 0.75 | 0.13 | 1.12 | 1.28 | 1.69 | 3.00 | 0.95 | 1.13 | 0.49 |
| **Lambayeque** | 0.53 | 0.26 | 2.01 | 0.45 | 0.07 | 0.99 | 0.39 | 0.64 | -0.68 | 1.72 | 3.12 | 1.20 | 0.80 | 0.30 |
| **Lima/Callao** | 0.18 | 0.13 | 0.77 | 0.17 | -1.49 | 0.68 | -0.19 | 0.59 | 0.47 | 0.79 | 0.56 | 0.95 | 0.10 | 0.25 |
| **Loreto** | 0.36 | 0.30 | 2.29 | 0.65 | -0.26 | 0.70 | 0.49 | 0.46 | -0.07 | 0.92 | 1.84 | 0.73 | 0.53 | 0.21 |
| **Madre de Dios** | 0.45 | 0.34 | 1.72 | 0.41 | -1.91 | 0.94 | -0.79 | 0.99 | -0.25 | 1.10 | 0.74 | 0.76 | 0.10 | 0.20 |
| **Moquegua** | -0.15 | 0.14 | 1.14 | 0.40 | 0.77 | 1.08 | 0.05 | 0.46 | -1.45 | 2.15 | 0.89 | 1.10 | 0.05 | 0.34 |
| **Pasco** | 0.02 | 0.23 | 2.76 | 0.46 | 0.11 | 0.88 | 0.05 | 0.71 | 0.72 | 1.00 | 1.29 | 0.98 | 0.67 | 0.39 |
| **Piura** | 0.25 | 0.14 | 1.90 | 0.43 | -1.15 | 0.59 | -0.72 | 0.43 | 0.90 | 1.45 | 0.42 | 1.43 | 0.20 | 0.29 |
| **Puno** | 0.60 | 0.28 | 2.33 | 0.54 | -0.78 | 0.91 | -0.48 | 0.90 | 1.16 | 1.33 | -0.99 | 1.55 | 0.61 | 0.33 |
| **San Martin** | 0.11 | 0.16 | 1.71 | 0.52 | -0.88 | 0.56 | -0.39 | 0.42 | 0.03 | 1.46 | 3.53 | 0.88 | 0.54 | 0.22 |
| **Tacna** | -0.22 | 0.21 | 0.55 | 0.27 | -1.75 | 0.75 | -0.34 | 0.66 | -2.61 | 1.86 | 1.04 | 1.38 | -0.32 | 0.25 |
| **Tumbes** | -0.17 | 0.23 | 0.61 | 0.26 | -0.60 | 0.38 | -0.15 | 0.40 | -0.85 | 1.85 | 0.30 | 1.75 | -0.09 | 0.32 |
| **Ucayali** | -0.01 | 0.32 | 1.54 | 0.64 | -1.63 | 0.72 | -0.77 | 0.65 | -0.67 | 0.85 | 1.77 | 1.02 | 0.29 | 0.42 |
